# Phosgene-Induced acute lung injury: Approaches for mechanism-based treatment strategies

**DOI:** 10.3389/fimmu.2022.917395

**Published:** 2022-08-02

**Authors:** Chao Cao, Lin Zhang, Jie Shen

**Affiliations:** ^1^ Research Center for Chemical Injury, Emergency and Critical Medicine of Fudan University, Shanghai, China; ^2^ Key Laboratory of Chemical Injury, Emergency and Critical Medicine of Shanghai Municipal Health Commission, Shanghai, China; ^3^ Center of Emergency and Critical Medicine, Jinshan Hospital of Fudan University, Shanghai, China; ^4^ Training Center of Acute Poisoning Treatment Technology of Fudan University Shanghai Medical College, Shanghai, China

**Keywords:** phosgene, lung, infection and lipids, acute respiratory distress syndrome, pulmonary edema, hypoxemia

## Abstract

Phosgene (COCl_2_) gas is a chemical intermediate of high-volume production with numerous industrial applications worldwide. Due to its high toxicity, accidental exposure to phosgene leads to various chemical injuries, primarily resulting in chemical-induced lung injury due to inhalation. Initially, the illness is mild and presents as coughing, chest tightness, and wheezing; however, within a few hours, symptoms progress to chronic respiratory depression, refractory pulmonary edema, dyspnea, and hypoxemia, which may contribute to acute respiratory distress syndrome or even death in severe cases. Despite rapid advances in medicine, effective treatments for phosgene-inhaled poisoning are lacking. Elucidating the pathophysiology and pathogenesis of acute inhalation toxicity caused by phosgene is necessary for the development of appropriate therapeutics. In this review, we discuss extant literature on relevant mechanisms and therapeutic strategies to highlight novel ideas for the treatment of phosgene-induced acute lung injury.

## Introduction

Phosgene is a type of poisonous gas that was initially used as a chemical weapon during World War I (1). It is widely applied in industrial processes, such as the synthesis of pesticides, plastics, dyes, polyurethanes, and metallurgy, and is indispensable in pharmaceutical production (2). Although phosgene is no longer used as a chemical weapon, phosgene-induced casualties still occur due to accidents resulting from improper operations. With rapid industrialization, approximately 12 million metric tons of phosgene are produced annually, and it is estimated that such production will rise to 18.6 million metric tons per year by 2030 (3). Due to the wide availability and usage of phosgene in chemical industries, numerous chemical and industrial employees, welders, and firefighters are at risk of exposure; therefore, phosgene exposure poses a significant health concern from both accidental and deliberate release. Nevertheless, specific medical treatments for the toxic effects of phosgene exposure are lacking. Accordingly, there is an urgent need to identify effective treatment strategies (4, 5).

Phosgene exposure predominantly damages the respiratory system. Phosgene-induced acute lung injury (P-ALI) is commonly associated with short-term phosgene inhalation (6, 7). Prolonged exposure can cause chronic hypoventilation, refractory pulmonary edema, and other associated lung injuries, ultimately resulting in acute respiratory distress syndrome (ARDS) (8, 9). Therefore, more attention should be paid to the high risk of mortality in critically ill patients. Following exposure to phosgene, the main symptoms initially include a mild dry cough, accompanied by skin or mucous membrane irritation in the early period (10). However, if the patient remains in the disease-causing environment or does not receive effective care, visual symptoms of respiratory irritation rapidly develop within a few hours, typically presenting as coughing, chest tightness, and wheezing (5). Following inhalation of mass amounts or prolonged inhalation, typical clinical symptoms of ALI, including pulmonary edema, dyspnea, and hypoxemia, are evident, which may progress into ARDS in severe cases (11). In the late stage of P-ALI, survivors often develop chronic lung disease with obstruction of airflow, fibrosis (12), airway hyperresponsiveness, and impairments in gas exchange (13, 14).

Statistically, deaths associated with phosgene exposure predominantly occur in the early stage of severe ALI; as these patients often require hospitalization (15, 16), numerous studies have focused on the acute phase. Studies of critical mechanisms in P-ALI have been conducted for nearly a century (17–19) but have failed to identify successful treatments for phosgene exposure. Therefore, most treatments are supportive rather than curative in nature, alleviating symptoms but not addressing the cause of the disease (20–22). Thus, exploring the pathogenesis of P-ALI is crucial for identifying effective therapeutics and treatment regimens. In this review, we discuss recent evidence concerning the pathophysiological mechanisms and therapeutic strategies for P-ALI, as well as potential applications, to provide a reference for the effective treatment of P-ALI.

## Characteristics of phosgene toxicity

Phosgene is a volatile, acidic, chloride-fuming liquid with a boiling point of 8.3°C (47°F) at standard temperature and pressure. At room temperature, phosgene exists in the form of gas. Phosgene has a molecular formula of COCl_2_ and is approximately 3.5 times denser than air (ρ = 3.5 g/mL), which facilitates its deposition in low-lying areas but impedes dissipation. The diffusion length of phosgene in an aqueous solution is approximately 8.8 μm, which is 4–8 times the thickness of the blood–air barrier (23). Phosgene is colorless at room temperature and smells of “rotten grass,” with unstable chemical properties. Thus, the lack of obvious symptoms and alarming odor characteristics exacerbates phosgene toxicity, as exposed individuals may not immediately be aware of their peril (24). This increases the probability that an individual may be exposed to potentially harmful concentrations prior to reaction. As dangerous exposure becomes evident only when phosgene inhalation is associated with severe symptoms, the morbidity rate of phosgene poisoning remains high.

P-ALI is characterized by toxic pulmonary edema after 6–24 h of exposure, and its severity is dependent on the concentration × exposure duration (C × t) (25). In this regard, the severity of edema is not solely determined by the concentration of inhaled phosgene, and chronic exposure to low concentrations may be worse than acute exposure to high concentrations (26). At a lower to moderate C (<50 ppm·min for dogs or rats), pulmonary edema will occur within 15–20 h after inhalation, a period typically described as the clinical asymptomatic latency or, more accurately, the clinical latency. Depending on C × t, the transition from asymptomatic pulmonary edema to potentially fatal pulmonary edema occurs precipitously within a few hours. At higher concentrations (>150 ppm·min for dogs or rats), phosgene exposure can lead to life-threatening and latent non-cardiogenic pulmonary edema (27, 28).

## Pathophysiological mechanisms of P-ALI

The mechanisms underscoring P-ALI are still poorly understood but have been suggested to include direct interaction and deterioration of lung surfactants resulting in impairment of the epithelial-endothelial barrier and changes in lung mechanics due to neuronal damage induced by free radicals (20), causing tissue destruction and mediator release (29). Phosgene is a low-water-soluble acylating agent, which inhibits its solubility in aqueous solutions lining the respiratory tract. Phosgene directly damages the respiratory tract and trachea (30) as it rapidly dissolves in amphiphilic fluids such as lung surfactants and reacts *via* acylation with nucleophilic moieties, causing irreversible changes in the cell membrane and intracellular structures. At sufficiently high concentrations, phosgene gas can harm the surfactant layer and deplete the surfactant activity of glutathione, contributing to elevated reactive oxygen species (ROS) generation and diffusion into the tissue layer, thereby impairing the tissue layer (e.g., epithelial cells, endothelial cells, neurons, and blood constituents) (31). Phosgene can undergo heterolysis and homolysis to form a highly reactive carbamoyl monochloride radical, leading to alteration and dysfunction of proteins and phospholipids and the production of harmful ROS and nitrogen species (4).

The alveolar surface is lined with a complex and highly surface-active substance, known as pulmonary surface-active substance, which consists of approximately 90% lipids and 5%–10% surfactant-specific proteins that protect the alveoli from collapse at the end of expiration by reducing surface tension (32). Phosphatidylglycerol is present at an unusually high percentage in the surfactant and can reflect the state of alveolar damage, where it primarily serves as a precursor for cardiolipin (33). Secreted phospholipase A2 of group IIA (sPLA2-IIA) is a crucial enzyme involved in the production of lyso-PC and fatty acids in the lung through surfactant phospholipid hydrolysis (34). On the other hand, given that surfactant phospholipids inhibit sPLA2-IIA expression by alveolar macrophages, hydrolysis of these phospholipids by sPLA2-IIA leads to the removal of sPLA2-IIA inhibition and, as a consequence, to the establishment of a vicious circle (35). Phosgene is a bifunctional electrophillic molecule that reacts with nucleophillic groups of cellular macromolecules such as phospholipids (36), proteins (37), and DNA (38). After phosgene inhalation, there may be a 1-h latency period, contributing to the degradation of lung surfactant, production of phospholipids metabolites, and infiltration of inflammatory cells into lung tissue (39). As phospholipid depletion continues, pulmonary permeability is impaired, causing continuous accumulation of high protein exudates within the lung, which eventually contributes to a permeability-type pulmonary edema (40). Furthermore, phosgene interferes with lipid peroxidation, which may also have an impact on the endothelium with multiple consequences on lung injury (4), and increased permeability could also disrupt the immune barrier, leading to further entry of pathogenic microorganisms into the host. The hydrophobicity of phosgene exacerbates its toxicity, often leading to delayed toxidrome, as the upper respiratory tract is moderately irritated. By the time symptoms appear, significant damage has occurred. Indeed, direct interactions of phosgene with the pulmonary surfactant contribute to atelectasis. This interaction is due to surfactant dysfunction and imbalances of Starling’s and Laplace’s laws (30) caused by increased interstitial pressure. Collectively, these events further destabilize the alveoli. Moreover, loss and depletion of phospholipids are important pathogenic mechanisms of P-ALI.

**Figure 1:**
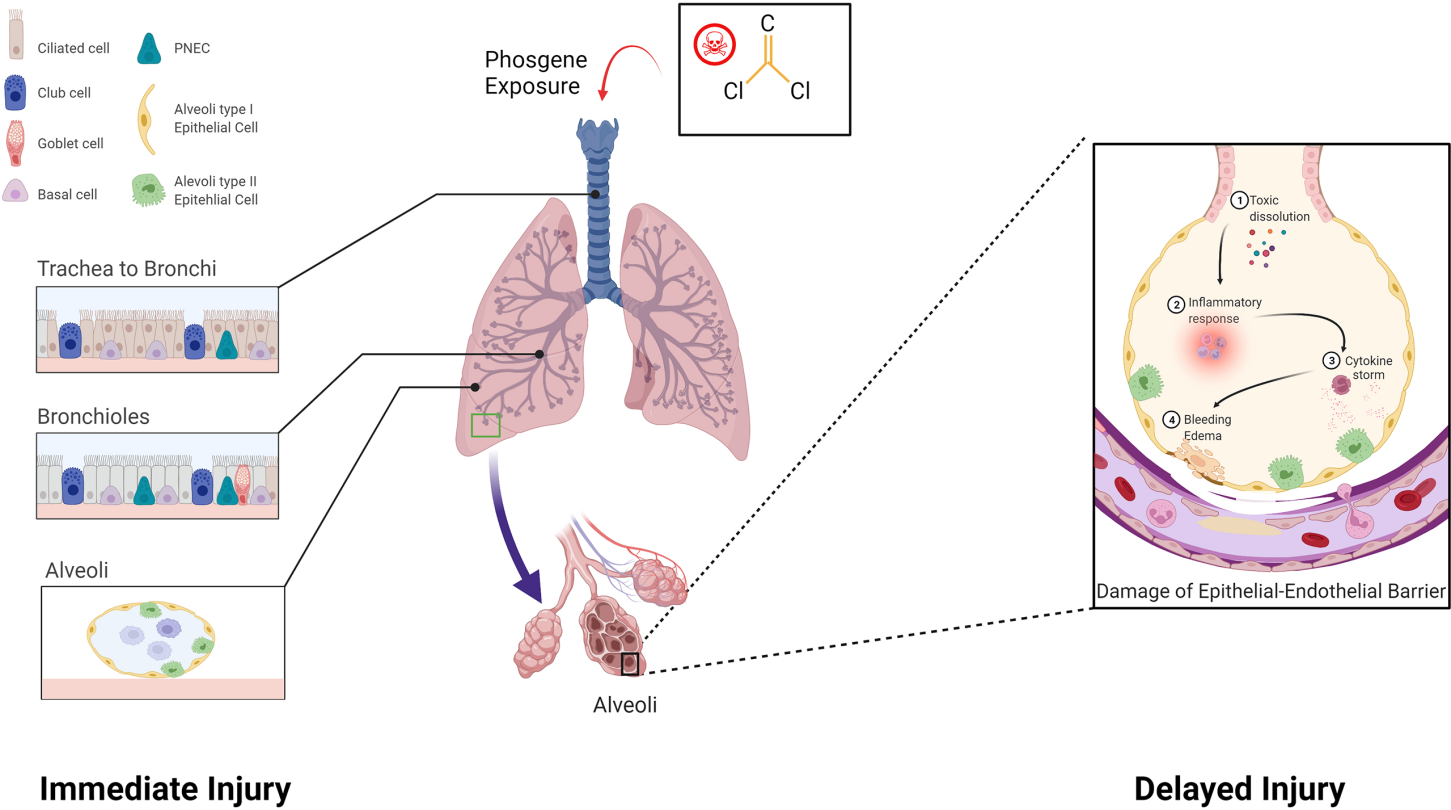
Pathological processes involved in phosgene-induced acute lung injury. Two pathological processes are involved: Immediate injury: Phosgene exposure damages the lungs, leading to necrosis of club cells and ciliated epithelial cells that cover the trachea, bronchus, and alveoli. Delayed injury: Uncontrolled inflammatory responses result in a cytokine storm, which leads to a cascade-like inflammatory reaction, secondary biological attack, and further aggravation of the epithelial-endothelial barrier (created with www.biorender.com).

It is generally acknowledged that two pathological processes are involved in P-ALI, including primary lung injury (direct injury to the lung air-blood barrier) and secondary lung injury (consequential inflammatory reactions) (41) (**Figure 1**). In the early stage of inhalation, phosgene-induced acylation of surfactants leads to surfactant depletion and exhaustion. Phosgene then reacts with the proteins, lipids, and nucleic acids in the alveolar tissue, thereby increasing alveolar surface tension and resulting in atelectasis (42). This eventually damages endothelial, epithelial, and innate immune cells, causing inflammation and epithelial-endothelial barrier dysfunction (42, 43). In the late stage, the disease progresses to refractory hypoxemia, pulmonary inflammatory infiltration, diffuse hemorrhage, and edema. The uncontrolled inflammatory reaction eventually leads to inflammatory waterfall-like changes in the lungs, resulting in ARDS (44, 45). In addition, phosgene can cross the blood-gas barrier and enter the capillary circulation, subsequently damaging red blood cells (RBCs). Increased fragility of RBCs, oxidative damage to RBC membranes, injury to plasma plasmalogens, and the onset of delayed but severe lung injury have been observed in mouse models of P-ALI (43).

## Cellular and molecular mechanisms of P-ALI

**Figure 2:**
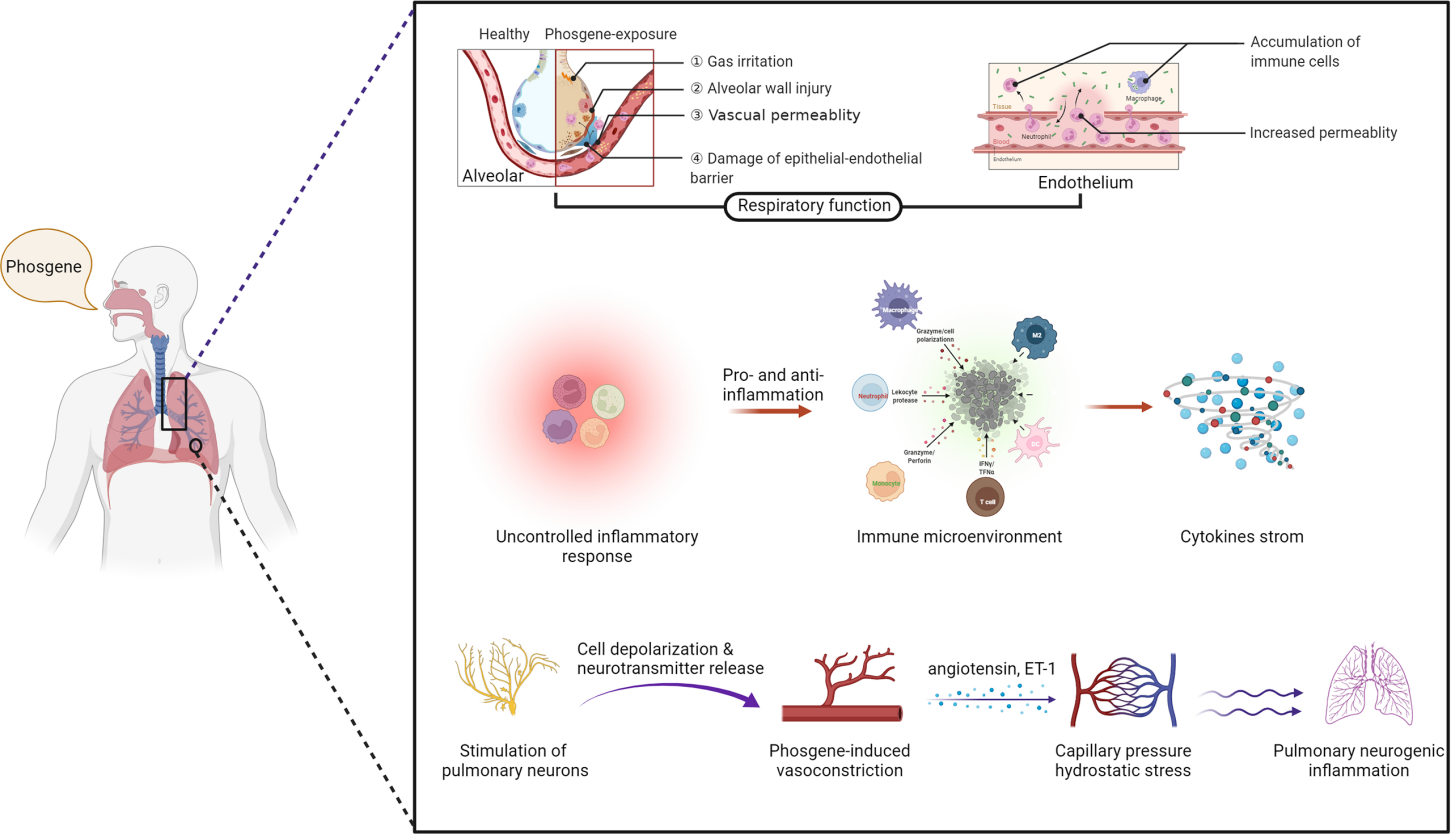
The mechanisms of phosgene-induced acute lung injury. Damage to the epithelial–endothelial barrier (upper layer), alteration of the immune microenvironment (middle layer) and stimulation of pulmonary neurons (bottom layer) (created with www.biorender.com).

The precise cellular and molecular mechanisms underlying P-ALI are complex processes that have yet to be fully elucidated. In this regard, various cell types contribute to the occurrence and/or progression of P-ALI, including immune cells, epithelial cells, endothelial cells, and pulmonary neurons (**Figure 2**).

### Damage to the epithelial–endothelial barrier

The biophysical structure of the host pulmonary defense response is intrinsically related to the ability of alveolar epithelial and capillary endothelial cells to form a barrier (referred to as the epithelial–endothelial barrier), which helps to regulate the immune response and protects the lungs against injuries and infections. Inhalation of phosgene leads to lesions of the pulmonary alveolus–capillary membrane (epithelial–endothelial barrier), which increases permeability to liquids and solutes, causing aggregation of liquid in the pulmonary alveoli and interstitium. This results in severe hypoxemia with key pathological and physiological changes such as osmotic pulmonary edema (27). The incipient pathogenesis of phosgene-induced ALI/ARDS commences with the loss of surfactant function, which is induced by a direct chemical reaction between surfactants and phosphine (46), especially in epithelial and endothelial cells. Phosgene can also react with water in the lungs to form hydrochloric acid and carbon dioxide or undergo acylation reactions, or with amino (-NH2), hydroxyl (-OH) and sulfhydryl (-SH) groups in acylation reactions. These chemical processes produce chloride derivatives that can react with proteins in the pulmonary alveoli and disrupt the blood-air barrier, leading to ALI or ARDS (7).

**Figure 3:**
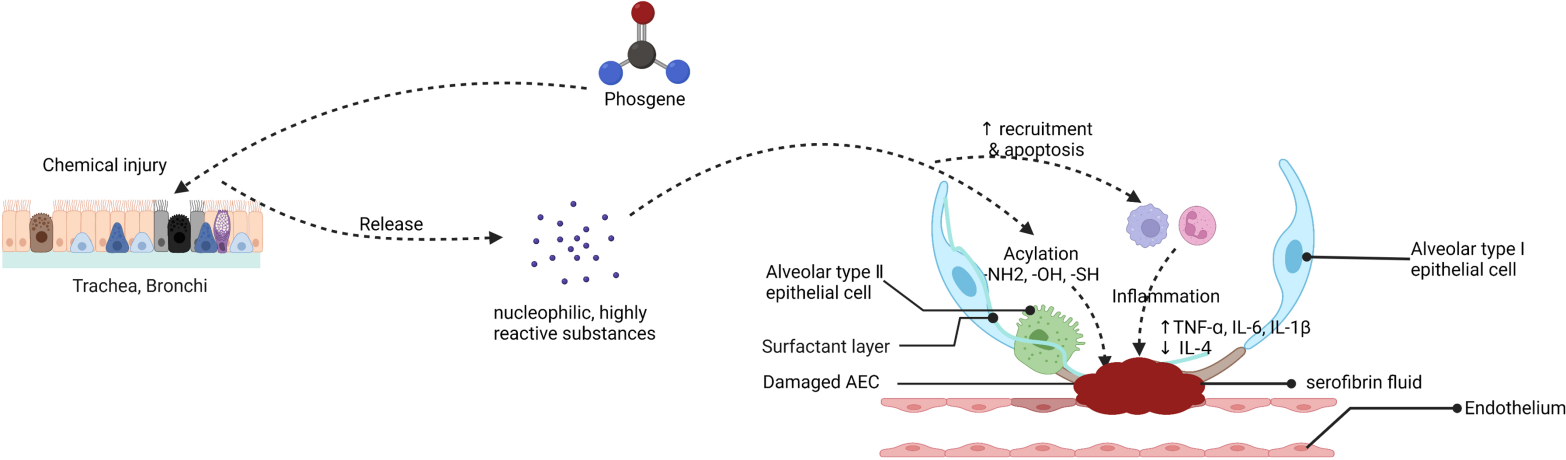
Putative mechanism of the endothelial-epithelial barrier damaged in phosgene-induced acute lung injury (created with www.biorender.com).

**Figure 4:**
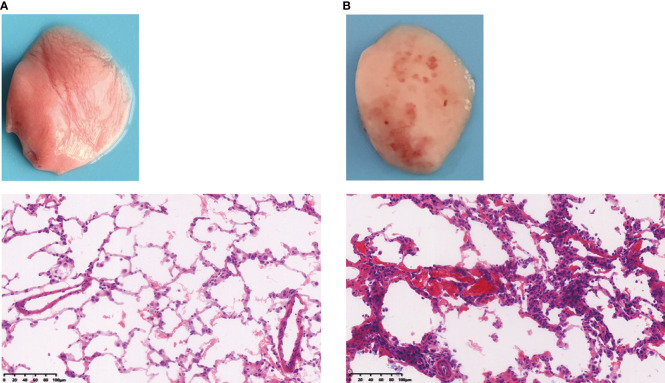
Rat lung tissues were compared to assess the difference between **(A)** healthy and **(B)** phosgene-induced acute lung injury after 24 hours of exposure. Rats were euthanized under ether anesthesia, and lung tissue from each experimental group was processed for histopathological evaluation (×100).

These disorders are characterized by endothelial and epithelial cell damage, surfactant dysfunction, loss of alveolar epithelial barrier integrity, and destruction of epithelial tight junctions. Upon phosgene inhalation, chemical processes produce large amounts of nucleophilic, highly reactive substances that infiltrate the alveoli, resulting in transient destabilization of alveolar surfactant and abnormal tension and collapse (47). This affects the function of immune cells, particularly macrophages, such as immune surveillance, cellular debris, and clearance of apoptosis after excessive surfactant depletion, stimulating massive recruitment and apoptosis of macrophages and neutrophils, and apoptotic immune cells may be secondary to necrosis, which could exacerbate lung injury. Furthermore, an imbalance in Starling forces composed of the colloid oncotic pressure and the hydrostatic hemodynamic pressure leads to partial infiltration of the lung’s alveolar and interstitial spaces, resulting in hyperpermeable (high surface tension) pulmonary edema (41, 48). Other studies have suggested that the mechanism of phosgene injury and increased vascular permeability is possibly caused by oxidative damage, associated with oxidants and arachidonic acid production (49). With increasing exposure time, leakage of serofibrin fluid increases and fills the alveolar space and extends into the bronchi and bronchioles and widens the perivascular space (**Figure 3**). Collectively, these mechanisms exacerbate alveolar epithelial permeability and lead to massive pulmonary edema, pulmonary fibrosis, or even death in some cases (50, 51). The most prominent histological changes are observed in alveolar epithelial, capillary endothelial, and adjacent interstitial tissues (27, 52) (**Figure 4**). Proteomic analysis and transmission electron microscopy have revealed that phosgene inhalation causes global proteomic modulations and marked ultrastructural changes in both alveolar epithelial cells (53) and capillary endothelial cells (54) in co-culture systems.

Moreover, a high concentration of phosgene can penetrate the lung surface and enter the active layers of the alveoli to deplete glutathione, leading to increased production of ROS, which can diffuse into the tissue layers and damage deeper cells (44). In summary, phosgene toxicity in human alveolar epithelial and capillary endothelial cells can elicit a significant immune response and lead to clinical disorders, causing immediate symptoms in the early stages of P-ALI.

### Alteration of the immune microenvironment

After the initial phosphine-mediated damage, there is a large influx of inflammatory cells into the lungs, which may exacerbate the damage (55), especially after the impairment of the epithelial-endothelial barrier. P-ALI is a life-threatening syndrome characterized by an explosive cascade of inflammation in the lungs. Under these conditions, interleukin-1β (IL-1β) and tumor necrosis factor-α (TNF-α) levels are several times higher after phosgene inhalation than under normal physiological conditions (56). Other inflammatory factors such as IL-4, IL-6, IL-8, and IL-10 are also involved in cellular events and play essential roles in phosgene-induced pulmonary injury. In previous studies, we examined alterations in pro- and anti-inflammatory cytokines and observed that decreasing inflammatory cytokine production and neutrophil accumulation reduced phosgene-induced ALI pathogenesis (52, 57). Of note, TNF-ɑ, IL-6 and IL-1β in both the serum and BALF were significantly upregulated, while anti-inflammatory cytokine IL-4 was decreased in P-ALI, which indicated these above inflammatory factors may play a key role in the pathology of P-ALI (56). In summary, anti-inflammatory treatment may represent a novel therapeutic modality for phosgene-induced ALI.

In the early stages of lung injury, extensive changes in immune cells alter the regulation of the immune microenvironment. Some phagocytic cells, such as heterophil granulocytes, accumulate and release toxic products, including oxyradicals (58). These toxic products, released by necrotic tissue and polymorphonuclear cells, can cause bronchoconstriction and exacerbate hypoxia. In a model of phosgene-induced lung injury, mechanisms of innate immune defense, such as the formation of neutrophil extracellular traps and histone release, can lead to more severe alveolar injury. The alteration of the immune system also generates ROS, leukocyte proteases, chemokines, and cytokines, which are effective against pathogens but may worsen the alveolar injury. These indicate that alterations in the immune environment may affect the process or prognosis of P-ALI or ARDS. Collectively, these findings suggest that anti-inflammatory therapy may be considered a therapeutic approach for the treatment of P-ALI.

### Stimulation of pulmonary neurons

Pulmonary neurogenic inflammation occurs when pulmonary neurons release inflammatory substances. Neurogenic pulmonary edema has been confirmed as a key causative factor of P-ALI. Following exposure, nociception and protection are achieved by activating afferent nerve fibers innervating the whistling tract. Sensory nerve endings are widely distributed on these afferent nerve fibers at all levels of the airway wall. Rothlin recognized that phosgene-induced pulmonary edema is caused by a reflex-mediated vascular response and was the first to hypothesize vagal neurogenic pathogenesis for such edema (59). Vagal C-fibers constitute the majority of vagal afferents innervating the lower respiratory tract (60). Under sufficient concentrations, phosgene can penetrate the tissue layer and stimulate pulmonary neurons, which subsequently react with key cellular components of epithelial and endothelial cells. Phosgene can directly or indirectly stimulate Ca^2+^ channels in pulmonary neurons, contributing to cell depolarization and neurotransmitter release, leading to vascular smooth muscle cell constriction and early phosgene-induced vasoconstriction. Concurrent with neuronal stimulation, phosgene-induced ROS form potent mediators (leukotriene, angiotensinogen and endothelin-1) that stimulate epithelial and endothelial cells to induce and release vascular tension and vascular permeability, promoting both short- and long-term microvascular contraction (4). This leads to increased capillary pressure, hydrostatic stress, and fulminant pulmonary edema. Neuronal-induced changes often precede pathological changes in studies of pulmonary edema. Inhalation exposure to phosgene can result in typical pulmonary vagal C-fiber stimulation symptoms, such as apnea, bradycardia, or cholinergic symptoms (46, 61). Therefore, the mechanisms of phosgene-induced pulmonary responses (i.e., vasoconstriction) may be mediated by neurogenic stimulation.

## Treatment

Despite the widespread industrial use of phosgene, there are currently no FDA-licensed therapeutics or evidence-based treatment guidelines for managing exposed individuals (4, 29, 62). At present, the therapeutic approach to P-ALI focuses on ameliorating clinical symptoms, including oxygen therapy, mechanical ventilation, aggressive pulmonary toilets, and avoidance of circulatory volume overload. Current treatments only improve lung ventilation but cannot improve or reverse pathological processes or lung injury. Although multiple studies have employed animal models, identifying active prevention strategies during the early stages of exposure is essential.

### Oxygen inhalation and mechanical ventilation therapy

The main life-threatening factors associated with phosgene-induced ALI and ARDS are oxygen deficiency and asphyxia. Oxygen therapy improved pulmonary pathology when administered at any concentration and time delay and was therefore extensively promoted as a supportive treatment in the early days of P-ALI, especially during World War I (63). However, growing evidence suggests that increasing oxygen exposure in tissues above normal levels may lead to the production of detrimental ROS, which may be more harmful, especially in the early asymptomatic phase of phosgene poisoning (64). Therefore, multiple studies have focused on different oxygen-delivery protocols.

Currently, it is well accepted that chronic high-flow oxygen damages the lungs. Reduced inhaled oxygen for short periods (up to 24 h) following ALI may contribute to further physiological deterioration, thereby increasing pulmonary edema, reducing arterial oxygenation, and worsening survival. However, it is recommended to delay inhalation of oxygen for P-ALI until signs or symptoms of hypoxia or a decrease in arterial oxygenation occur. In this regard, the minimum oxygen concentration that maintains normal arterial oxygen saturation without leading to clinical signs of hypoxia is considered beneficial (21). Intravenous infusion of a hyper-oxygenated solution has been reported to attenuate ALI by ameliorating the formation of lung edema, lipid peroxidation, and hypoxemia associated with phosgenismus, thereby highlighting a novel protective strategy against ALI (65).

Other measures, such as increasing the fractional concentration of inspired oxygen (FiO_2_), using positive end-expiratory pressure (PEEP), or physical rest, have been suggested as treatments for chemically induced ALI and ARDS (66). Parkhouse et al. reported improved survival using protective ventilation strategies incorporating PEEP following phosgene exposure. This approach may improve arterial oxygenation in pulmonary edema by recruiting alveoli, redistributing lung fluid, and opening and stabilizing atelectatic alveoli (67). Another study reported that ambient air continuous positive airway pressure (CPAP) support could improve survival and ameliorate clinically relevant physiological changes associated with P-ALI (e.g., changes in arterial oxygenation and respiratory rate) (22). Although these tools cannot alleviate the progression of lung injury, mechanical ventilation plays an indispensable role in the supportive treatment of P-ALI.

Clinicians should avoid unreasonable or common symptomatic treatments that may accelerate P-ALI progression. Preventative and individualized treatment strategies addressing feedback circulation and mechanical ventilation based on lung function should be prioritized, along with conservative fluid management.

### Pharmacological treatments

Multiple approaches for drug-related interventions have been explored in experimental research, most of which involve anti-inflammatory and sympathomimetics. Nevertheless, almost none of these treatments have been implemented clinically (5, 68, 69). Following initial phosgene-mediated lung damage, inflammatory cells rapidly enter the lungs, which may aggravate lung damage (70). Therefore, many guidelines suggest that glucocorticoids should be administered as an indispensable anti-inflammatory treatment. Glucocorticoids are widely used in managing ALI due to their rapid and powerful (but nonspecific) anti-inflammatory effects. However, the use of glucocorticoids in phosgene-poisoning treatment remains controversial. It is generally concluded that short-term administration may effectively alleviate damage to the lung endothelium and promote the absorption of glucocorticoids in pulmonary edema. These anti-inflammatory effects may be exerted *via* several mechanisms, such as inhibiting the release of inflammatory cytokines, neutrophil activation, reducing capillary permeability, and promoting the alveolar macrophage differentiation into the M2 phenotype (44). However, given the scope of usage, the adverse effects and side effects of long-term application should be considered. Moreover, these approaches have even been reported to be ineffective for P-ALI (71, 72). Exploratory preclinical evidence has identified other novel pharmaceutical approaches for preventing P-ALI, such as neuro-regulators, calcium regulators, antioxidants, and endothelin receptor antagonists (7, 57, 73–85), which warrant further investigation (**Table 1**). To some extent, these studies have positively affected treatment, which provided new perspectives for a deeper understanding of P-ALI pathogenesis.

**Table 1:** Summary of the effects and mechanisms of pharmacological-mediated therapy the treatment of P-ALI.

Medication	Mechanism of action	Route	Species	Refs.
NOS-2 Inhibitors	Preserved epithelial integrity by attenuating the reduction in ZO-1 expression and augmenting expression of SP-B.	Aerosolized inhalation	Mouse	7
NLRP3	Inhibiting NLRP3 inflammasome activation and pro-inflammatory factors.	Intravenous injection	Rat	73
Angiopoietin-1	Attenuation of inflammatory response.	Intravenous injection	Rat	57, 74, 75
Melatonin with Ulinastatin	Improved pulmonary edema and attenuated pulmonary inflammation via Wnt/β-catenin pathway.	Intraperitoneal injection	Rat	76
Ulinastatin	Decreased the infiltration of blood cells and reduces inflammatory cytokines.	Intraperitoneal injection	Rat	77
N-Acetylcysteine	Protected against oxidative stress through acting on Nrf2/GR/GSH pathway.	Intraperitoneal injection	Rat	78
Pentoxifylline	Inhibited ICAM-1 differential expression and improved inflammation.	Intraperitoneal injection	Rat	79
CAPE	Antioxidant and anti-inflammatory function via blocking translocation of NF-κB p65 to nucleus.	Intraperitoneal injection	Rat	80
Aminophylline	Decreased pulmonary capillary permeability and attenuated lipid peroxidation.	Intravenous injection	Rabbit	81
IBU with PTX	Alleviated pulmonary edema and decreased inflammatory responses .	Intraperitoneal injection	Rat	82
DBcAMP	Alleviated pulmonary endothelial or epithelial cell contraction via antioxidant effect.	Intratracheal injection	Rabbit	83
nPG with Vitamin E	Decreasing lipid peroxidation and increasing lung tissue glutathione.	Oral	Mouse	84
Colchicine	Improved respiratory function by diminishing the incursion of inflammatory cells.	Intraperitoneal injection	Rat	85

NOS-2, nitric oxide synthase 2; NLRP3, NOD-like receptor protein 3; CAPE, caffeic acid phenethyl ester; IBU, ibuprofen; PTX, pentoxifylline; DBcAMP, post-treatment with dibutyryl cAMP; nPG, n-propyl gallate.

Inflammation and oxidative stress are key mechanisms underscoring sublethal phosgene injury. Most pharmacological treatments focus on anti-inflammatory or antioxidant therapy; therefore, exploring positive prevention strategies in the early stages of exposure is advisable. Although all positive data have traditionally been based on hypothetical pathways clarified by rodent *in vivo* studies, these studies provide novel perspectives for clinical application.

### Mesenchymal Stem Cells (MSCs)

MSCs are non-hematopoietic stem cells with multipotent differentiation potential in the bone marrow. As a type of heterogeneous pluripotent progenitor cell, MSCs possess strong immunoregulatory abilities and aid in the maintenance and regeneration of various cell types by differentiating into multiple lineages of mesenchymal tissues. Therefore, MSCs have been introduced as a potential treatment for ALI and other pneumonia-associated diseases (86, 87). MSCs redistribute to inflammatory tissues and organs (88, 89) and play a protective role by targeting the lesion location, adopting an epithelium-like phenotype, and alleviating inflammation and collagen deposition during ALI and ARDS (90, 91). In addition to immunomodulatory effects, MSCs also differentiate into pulmonary epithelial cells and endothelial cells, which function in the repair of the blood barrier and blood gas recovery (92).

In 2015, our research team was the first to verify the therapeutic effects of MSCs in P-ALI. Mechanistically, this process is involved in pulmonary air-blood barrier repair and regulating inflammatory reactions (93). Further studies have confirmed the related mechanisms of action from various aspects, including regulation of the proliferation and differentiation of lung epithelial cells and endogenous lung stem cells (56) and repair *via* the proliferation of endogenous lung stem cells (94). Although most studies focusing on experimental models have demonstrated their validity, MSCs provide strong prospects for treating P-ALI (6, 52, 53, 94–98) (**Table 2**). MSC-based therapy has been widely used in the clinical treatment stage of ARDS and has also achieved very good effects, and it is expected to be studied in the P-ALI clinic in the near future.

**Table 2:** Summary of MSCs-mediated therapy used *in vivo* in the treatment of P-ALI.

Therapeutic approach	Outcomes	Mechanism	Refs.
MSC-derived exosomes, intratracheally injection	Adjusted indexes of respiratory function;	Ameliorated respiratory function through suppressing matrix metalloproteinase-9 synthesis, and improving synthesis of SP-C.	6
	Reduced TNF-α, IL-1β and IL-6, but increased IL-10.		
Exogenous MSCs, intravenous treatments	Reduced epithelial permeability and disruption of tight junction protein in phosgene-exposed lung.	Homed to sites of lung injury, reduced epithelial permeability likely by blocking wnt3/β-catenin signaling.	52
MSCs transfected with CXCR7, via tail vein	Improved pulmonary histopathology and repaired tissue;	Promoted differentiation into AT II cells and alleviated the	53
	Attenuated pulmonary inflammation.	lung inflammation more effectively.	
Exogenous MSCs, intraperitoneally injected	Enhanced the proliferation of club cells, promoted lung injury repair.	Enhanced the proliferation of club cells partly via activating Notch signaling pathway.	94
MSCs over-expression of heat shock protein (HSP) 70, trachea administration	Regulated MSCs antiapoptotic and migration ability;	Enhanced MSCs viability through the PI3k/AKT mediated signaling pathway.	95
	Reduced TNF-α, up-regulated IL-10.		
MSCs transfected with miRNA-378a-5p, tracheal infusion	More effective by repairing alveolar epithelial cells;	Restored respiratory indexes and regulated pro- and anti-inflammatory response.	96
	Improving permeability of vascular endothelial cells compared with MSCs alone.		
Angiopoietin-1 infected into MSCs, via tail vein	Increased level of epithelial cell marker in lung tissues;	Angiopoietin-1 facilitated homing of MSCs to injure and repaired epithelial tissue.	97
	Regulated pro- and anti-inflammatory response.		
MSCs transfected with HSP 60, tracheal infusion	Alleviated pulmonary edema, regulated inflammatory responses and immune microenvironment.	Enhanced the ability of proliferation, anti-apoptosis, migration and the curative effect of MSCs.	98

MSC, mesenchymal stem cell; HSP, heat shock protein; TNF, tumor necrosis factor; IL, interleukin.

Several issues limit the widespread use of MSCs in clinical practice. The low homing efficiency of intravenous MSCs is an obvious problem (99), and the survival rate of MSCs after entering the body should be considered. In addition, MSC injection has been associated with a high risk of thrombosis and embolism (100), and separation and purification technology are currently unable to satisfy the requirements of large-scale clinical application (101). Collectively, these factors hinder the transformation from experimental research to clinical practice.

It is important to note that the P-ALI treatment is focused on reducing direct damage to the trachea, bronchi, and alveoli from irritating gases in the early stages of the disease. With the progression, the therapeutic direction should be focused on ameliorating inflammatory response, regulating immune microenvironment and damaged tissue repair. Thus, MSC-based treatment offers very promising therapeutic prospects for improved targeted therapies. Future preclinical and clinical trials are essential to evaluate and optimize therapies to best address the ongoing challenges in translational MSCs research.

## Conclusions

Phosgene is a widely used but dangerous chemical product that can cause serious lung injury, resulting in immediate toxic effects and incapacitation after unexpected exposure. Nevertheless, the toxicity of phosgene remains understudied, and its mechanisms of action remain obscure. To explore new therapeutic approaches and identify approved drugs that can be harnessed to treat P-ALI, a deeper understanding of the mechanisms of injury, including those at the cellular level, is required. More in-depth research is warranted to understand better the underlying mechanisms that will provide survival benefits and can be extrapolated to humans. Despite considerable progress, a single therapeutic seems unlikely to be sufficient for improving the various direct and secondary toxic effects of phosgene inhalation.

## Data availability statement

The original contributions presented in the study are included in the article/supplementary material. Further inquiries can be directed to the corresponding author.

## Author contributions

CC prepared figures and contributed to the writing of the manuscript. LZ participated in the writing of the manuscript. JS designed and contributed to the writing of the manuscript. All authors agree to be accountable for the content of the work.

## Funding

This work was supported by grants from the National Natural Science Foundation of China (Grant No. 81902007 to CC), the Natural Science Foundation of Tianjin (Grant No. 19JCQNJC10000 to CC), Binhai New District Health Commission Science Foundation of Tianjin (Grant No. 2019BWKY012 to CC), and the Science and Technology Committee of Jinshan District, Shanghai (Grant No. 2019-3-07 to JS).

## Acknowledgments

The authors thank the Laboratory Center of Jinshan Hospital for excellent technology support.

## Conflict of interest

The authors declare that the research was conducted without any commercial or financial relationships that could be construed as a potential conflict of interest.

## Publisher’s note

All claims expressed in this article are solely those of the authors and do not necessarily represent those of their affiliated organizations, or those of the publisher, the editors and the reviewers. Any product that may be evaluated in this article, or claim that may be made by its manufacturer, is not guaranteed or endorsed by the publisher.
